# Nickel-Assisted Laser Oxidation of WSe_2_ Layered Films for Spatially Controlled WO_3_ Patterning Process toward Resistive Memory and Molecular Sensing

**DOI:** 10.1021/acsami.5c17500

**Published:** 2025-12-01

**Authors:** Yu-Chieh Hsu, Ruei-Hong Cyu, Yu-Qi Huang, Chieh-Ting Chen, Po-Chien Lai, Yi-Jen Yu, Chang-Hong Shen, Yu-Lun Chueh

**Affiliations:** † Department of Materials Science and Engineering, 34881 National Tsing-Hua University, Hsinchu 30013, Taiwan; ‡ College of Semiconductor Research, National Tsing-Hua University, Hsinchu 30013, Taiwan; § Department of Physics, National Sun Yat-Sen University, Kaohsiung 80424, Taiwan; ∥ Department of Materials Science and Engineering, Korea University, Seoul 02841, Republic of Korea; ⊥ Instrument Center, National Tsing Hua University, Hsinchu 30013, Taiwan

**Keywords:** laser oxidized WO_3_, patternable laser process, WSe_2_ and WO_3_ based RRAM, SERS measurements

## Abstract

A novel approach to convert a 2D WSe_2_ film into a WO_*x*_ film by the laser-induced oxidation process using a nickel (Ni) adsorption layer, namely Ni-assisted laser oxidation process, was demonstrated. A layer of Ni metal was deposited to absorb the heat of the laser and oxidize the top WSe_2_ layer, for which an aluminum oxide (Al_2_O_3_) layer is then deposited as a barrier layer. A continuous wave laser with a wavelength of 808 nm is selected as the laser source, as it is not absorbed by the WSe_2_ layer. By introducing a patterned Ni layer, the selective oxidation process on WSe_2_ into WO_3_ can be achieved owing to the photothermal effects caused by the Ni layer with O_2_ gas. The successful oxidation parameters, including laser powers and irradiation durations with a fixed Al_2_O_3_ barrier layer thickness of 50 nm, were investigated. Resistive random-access memory (RRAM) devices fabricated using the Ni-assisted laser-oxidized WO_3_ structure exhibit clear resistive switching behavior compared to the structure without the laser oxidation process. In addition, the Ni-assisted laser-oxidized WO_3_ structure showed obvious surface enhanced Raman spectroscopy (SERS) signals of crystal violet (CV) and methylene blue (MB) with the lowest concentration of 10^–6^ M.

## Graphical abstract

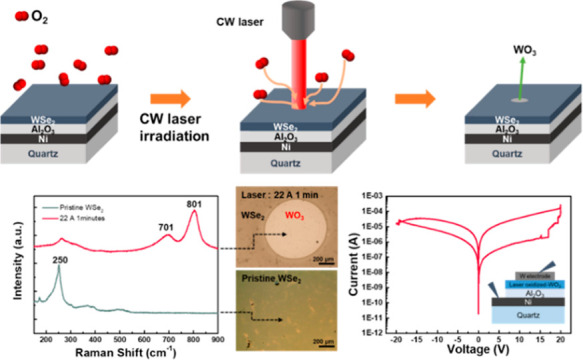

## Introduction

Two-dimensional (2D) transition metal dichalcogenides (TMDCs) have attracted extensive interest due to their unique electrical, optical, and catalytic properties.^1–3^ Unlike graphene, which lacks a bandgap, TMDCs such as MoS_2_, WS_2_, and WSe_2_ exhibit thickness-dependent semiconducting properties with bandgaps ranging from 1 to 2 eV, making them suitable for field-effect transistors (FETs), photodetectors, and memory devices.^4–7^ Their atomic thickness, flexibility, and strong light–matter interaction enable efficient scaling and integration in van der Waals heterostructures. Among various TMDCs, tungsten diselenide (WSe_2_) has emerged as a remarkably versatile candidate due to its ambipolar carrier transport, high mobility, and stability under ambient conditions.^8–10^ These features make WSe_2_ attractive for both digital and analog applications, including complementary logic circuits and neuromorphic architectures.^11–13^ Moreover, the methods to modulate its properties through chemical doping, phase engineering, and localized oxidation have opened up new routes for tailoring in-plane heterojunctions and functional interfaces.^14–16^ Despite these advantages, the scalable and spatially controllable patterning of 2D WSe_2_ layered films into different heterostructures remains a significant challenge. In particular, converting 2D WSe_2_ layered films into tungsten trioxide (WO_3_) films with high precision and compatibility with existing device fabrication processes are still under development.

The precisely controlled oxidation of 2D WSe_2_ layed film has proven to be an effective strategy to modulate its electrical and chemical properties, enabling applications ranging from logic transistors to neuromorphic devices.^17–19^ For instance, self-limiting thermal oxidation has been shown to selectively convert an atomically thin 2D WSe_2_ layer into an ultrathin WO_3_ film while preserving lateral resolution, facilitating in-plane heterojunction engineering.^18,20^ Likewise, oxidized WSe_2_-based FETs have demonstrated improved performance because of well-defined interfaces between semiconducting and insulating regions.^21^ Among all oxidation techniques, a conventional laser-assisted oxidation process provides a rapid and mask-free route to locally transform WSe_2_ into WO_3_ using a focused laser beam irradiated under an ambient atmosphere.^22^ However, the conventional laser oxidation often suffers from limited spatial control due to thermal diffusion, resulting in poorly defined oxide boundaries. Additionally, the lack of site-selective activation limits its utility in the precise patterning process. Recent efforts have explored lateral WSe_2_/WO_3_ heterostructures for memristive and synaptic applications, underscoring the promise of such systems for neuromorphic electronics.^23^ Nevertheless, achieving deterministic and high-resolution heterointerfaces remains a technical barrier.

To address these limitations, we present a systematic investigation on the Ni-assisted laser oxidation process to convert TMDs into metal oxide systems, where 2D WSe_2_ films were chosen for the idea demonstration. By introducing a patterned Ni layer, the oxidation process can be selectively achieved at designated sites due to a photothermal effect caused by the Ni layer. This approach offers improved spatial selectivity and controllable oxidation compared to conventional laser oxidation, since the Ni layer acts as a localized heat absorber that confines the reaction region and minimizes unwanted damage to adjacent areas. Structural and compositional results using Raman spectroscopy, atomic force microscopy (AFM), transmission electron microscopy (TEM), and X-ray photoelectron spectroscopy (XPS) confirm the successful and localized formation of the WO_3_ films from the 2D WSe_2_ layered films by the Ni-assisted laser oxidation process. Then, the effects of laser parameters and oxygen atmosphere for the Ni-assisted laser oxidation process were investigated. Meanwhile, unlike previous studies that relied on blanket oxidation or uncontrolled spot irradiation, our method enables the formation of complex and well-resolved oxide patterns, such as the NTHU pattern, with clear chemical contrast. Moreover, RRAM devices based on the Ni-assisted laser-oxidized WO_3_ structure exhibit pronounced resistive switching behavior compared to those without laser oxidation. The observed on/off ratio is about 100 at an operating voltage of 1 V for the Ni/Al_2_O_3_/laser oxidized-WO_3_/W structure, while no switching behavior was observed for the Ni/Al_2_O_3_/WSe_2_/W structure without the Ni-assisted laser oxidation process and the Ni/Al_2_O_3_/WO_3_/W structure that the WO_3_ film was only deposited by E-beam evaporation. In addition, the same structure demonstrates clear SERS responses, showing distinct signals for both crystal violet (CV) and methylene blue (MB). This Ni-assisted laser oxidation strategy offers a scalable, mask-free method for spatially programmable oxide patterning in 2D materials. Our work provides a new direction for engineering 2D heterostructures through Ni-assisted, site-selective laser processing, with potential applications in memory devices and integrated molecular sensing platforms.

## Results and Discussion

Figure 1 illustrates the initial characterization of the WO_3_ film formed by the Ni-assisted laser oxidation process from 2D WSe_2_ layered films. This method highlights the role of the Ni adsorption layer in facilitating oxygen diffusion and reaction at the surface of the 2D WSe_2_ layered film. The detailed fabrication processes of the Ni (50 nm)/Al_2_O_3_ (50 nm)/WSe_2_ (40 nm) stacking and the laser oxidation process are shown in Figure S1a. Note that to avoid the reaction of Ni and WSe_2_ layered film, a Al_2_O_3_ film prepared by the E-gun deposition was used as the barrier layer, and the uniform WO_*x*_ film was deposited by E-gun after the deposition of the Al_2_O_3_ film. Figure S1b,c display an optical microscopy (OM) image and a Raman spectrum of the uniform WO_*x*_ film. Then, the 2D WSe_2_ layered films were synthesized by the plasma-assisted selenization process from the E-gun deposited WO_*x*_ film, for which the heating and cooling curves of the plasma-assisted selenization process are shown in Figure S1d.^24^ After that, the samples were loaded into a laser vacuum chamber, which can apply oxygen gas inside, to carry out the laser oxidation process. The continuous wave (CW) laser with a wavelength of 808 nm was used for the oxidation process. For the selection of metal as the laser absorption and heating layer, Ni layer has been demonstrated to exhibit the highest absorption behavior at a wavelength of 808 nm compared with other metals by the Beer–Lambert law.^25^ The detailed parameters for sample preparation and the laser oxidation process are described in the method section. To confirm that most of the laser will be absorbed by the Ni layer, we also compared the light absorption of Al_2_O_3_ and WSe_2_ films, as shown in Figure 1b. Ni still shows the highest absorbance above the Al_2_O_3_ and WSe_2_ layers at the wavelength of 808 nm, which indicates that the Ni layer will trigger the oxidation reaction on the WSe_2_ film with O_2_ gas to form a WO_3_ film by a thermal annealing process because of a photothermal effect on the Ni layer. Note that the function of the Al_2_O_3_ layer acts as a separation layer between the WSe_2_ and Ni layers. Therefore, the overall laser oxidation process is dominated by the underlying Ni layer, which is called the Ni-assisted laser oxidation process. The OM images and corresponding Raman spectra before and after Ni-assisted laser oxidation are provided in Figure 1c, revealing the successful oxidation of WSe_2_ into WO_*x*_. After the Ni-assisted laser oxidation process, there is an obvious color change with a circular pattern in the upper OM image, suggesting that the oxidation areas, namely the WO_3_ area. The Raman spectra confirm that the phase should be WO_3_, with which peaks at 701 and 801 cm^–1^ were measured at the laser-irradiated area, while Raman signals of the only WSe_2_ peak at ∼250 cm^–1^ appeared before the Ni-assisted laser oxidation process, corresponding to the OM image without any color difference.^26^ To confirm that the surface of the WSe_2_ film remained intact after the Ni-assisted laser oxidation process, an atomic force microscope (AFM) was used to compare the surface roughness before and after the Ni-assisted laser oxidation process, as shown in Figure 1d. The values of *R*_a_ before and after the Ni-assisted laser oxidation process are 0.53 and 0.54 nm, respectively, and the corresponding AFM images also show no significant difference on surface morphology, suggesting that the surface is not destroyed by the Ni-assisted laser oxidation process.

**Figure 1:**
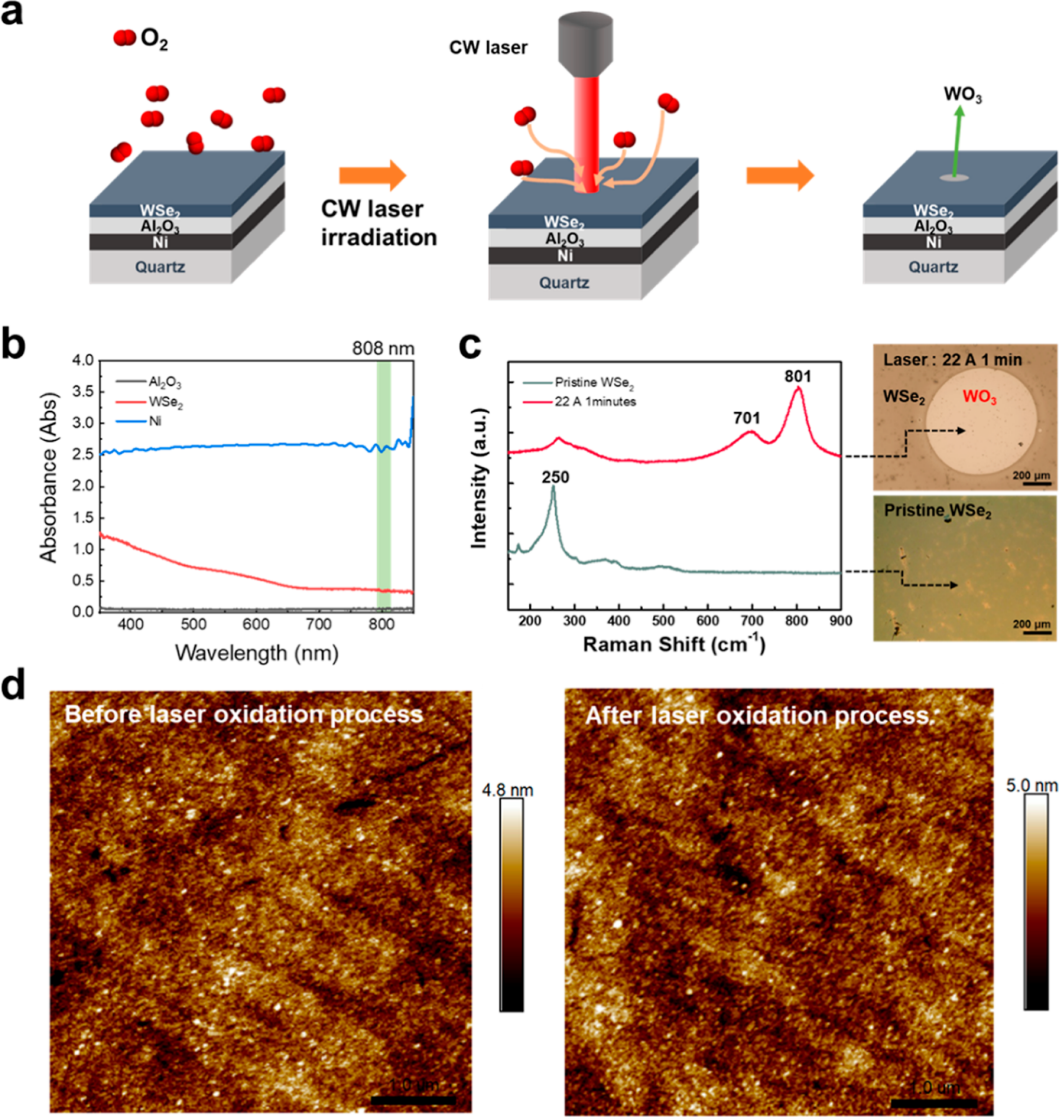
(a) Schematic diagram of WSe_2_ oxidation by the Ni-assisted laser oxidation process. (b) Absorption spectra of Ni, Al_2_O_3,_ and WSe_2_ films. (c) An Optical image and corresponding Raman spectra of the WSe_2_ film before and after the Ni-assisted laser oxidation process. (d) AFM images of the WSe_2_ film before and after the Ni-assisted laser oxidation process.

To gain deeper insight into the spatially resolved effects of the Ni-assisted laser oxidation process on the WSe_2_ film, detailed characterizations were performed. An OM image and corresponding Raman spectra from three distinct regions, including inside the laser-irradiated area (red spot), at the boundary of the laser-irradiated area (blue spot), and outside the laser-irradiated area (gray spot), are shown in Figure 2a. From the Raman spectra, strong WO_3_ Raman signals emerge inside the laser-irradiated area and the boundary, while pristine WSe_2_ peaks dominate the region outside the laser irradiation zone. Raman mapping images of the WO_3_ characteristic peak at 800 cm^–1^ at the center of the laser-irradiated area with an area of 100 × 100 μm^2^ are presented in Figure 2b, which shows uniform oxidation results within the laser-irradiated area. The uniform oxidation can be expected due to the uniform thermal heating provided by the underlying Ni layer, which significantly reduces the effect of the nonuniform radical distribution of the laser beam during the laser irradiation process. Furthermore, we investigate detailed parameters of the Ni-assisted laser oxidation, including laser power and laser irradiation durations (time), while fixing the thicknesses of WSe_2_, Al_2_O_3_, and Ni layers of 40, 50, and 50 nm under the O_2_ gas of 50 sccm, as plotted in Figure 2c. Note that OM and Raman results were utilized to determine all the results. The × patterns represent conditions without any reaction after the Ni-assisted laser oxidation process, revealing that the laser power is too low or the irradiation time is insufficient to trigger the oxidation reaction from WSe_2_ to WO_3_ film. The purple triangular patterns predominantly appear at laser parameters with high power and a damaged surface after applying a laser power of 23 W for 60 s, as shown in Figure S2a. The corresponding Raman spectra after applying the laser power of 23 W with the irradiation time of 60 s also show no material left at the center of the damaged surface, although the WSe_2_ could be successfully oxidized at the undamaged place (Figure S2b). Most importantly, the red star patterns represent suitable laser parameters that can successfully oxidize WSe_2_, as indicated by the Raman and OM results after the Ni-assisted laser oxidation process. Furthermore, it can be applied to the line-scan process from our CW laser system. The schematic diagram and corresponding parameter table are shown in Figure S2c, for which the Y-scale represents the scan rate of the laser and the X-scale represents the power of the laser. Based on the table, the laser power of 19 W with laser scan rates of 0.5 and 1 mm/s can achieve the oxidation of the WSe_2_ into the WO_3_, indicating that a higher laser power is needed for the line scan oxidation process than the point scan process. Apart from the laser scanning mode, we investigated the effect of the different Al_2_O_3_ thicknesses between the Ni and WSe_2_ layers. As the thickness of the Al_2_O_3_ oxide layer increases to 100 nm, longer irradiation time and higher laser power are needed to successfully oxidize the WSe_2_ into WO_3_, as shown in Figure S2d. The reason can be explained by the lower heat transfer efficiency from the Ni layer to WSe_2_.

**Figure 2:**
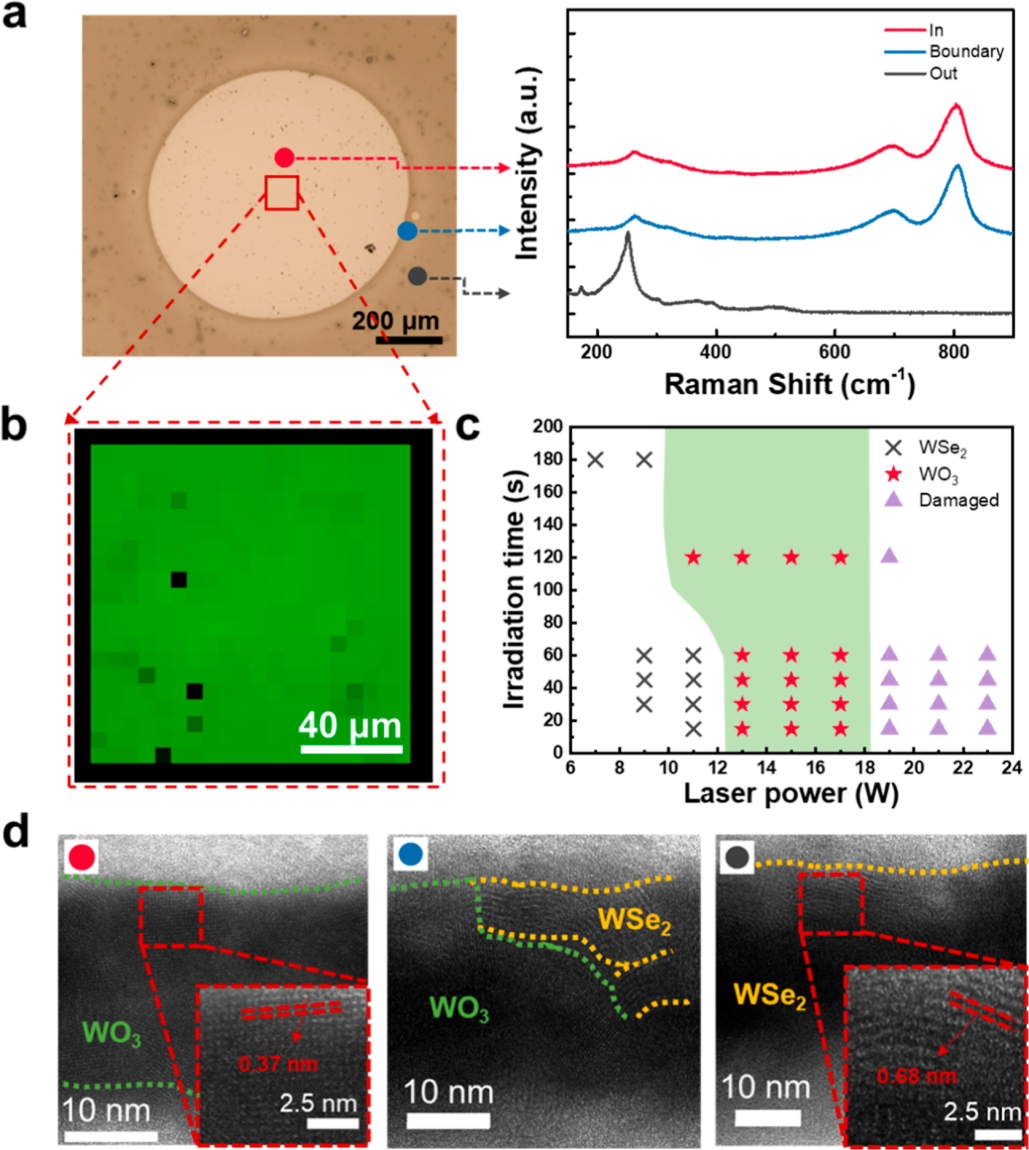
(a) Optical image and corresponding Raman spectra of the positions inside the laser spot, at the boundary of the laser spot, and outside the laser spot. (b) Raman mapping images of the WO_3_ peak at the laser annealing position. (c) The laser oxidation results were acquired under various laser powers and irradiation times, using a laser point. (d) Cross-section TEM images of positions inside the laser spot (red spot), at the boundary (blue spot), and outside the laser spot (gray spot).

Furthermore, the transmission electron microscopy (TEM) analysis was used to investigate the WSe_2_ and WO_3_ structures after the Ni-assisted laser oxidation process. The cross-sectional TEM analyses were carried out at three representative positions in the OM image of Figure 2a, including inside the laser spot (red spot), at the boundary (blue spot), and outside the irradiated area (gray spot), respectively (Figure 2d). In the center region, a distinct WO_3_ layer with a thickness of ∼30 nm with a crystalline structure was observed (Figure 2d1). The visible lattice spacing values are about 0.37 nm, which highly correspond to the crystallized structure of WO_3_.^27^ The boundary region at the blue spot site showed partial oxidation, with a mixed-phase structure of WO_3_ and residual WSe_2_. The WO_3_ phase appeared at the left-hand side, and the WSe_2_ phase is mainly observed on the right-hand side, according to the location of the blue spot in Figure 2a and d2. Moreover, the TEM image outside the irradiated area shows the crystalline structure of WSe_2_ with a lattice spacing value of 0.68 nm for the entire layer (Figure 2d3).^28^ Elemental analysis by TEM-energy dispersive spectroscopy (EDS) line scanning (Figure S3) further supported these structural transitions. The line scan profiles show no remaining Se after the Ni-assisted laser oxidation process, while the atomic ratio of W and O is about 30% and 70%, respectively.

Furthermore, X-ray photoelectron spectroscopy (XPS) was utilized to analyze the composition of the samples before and after the Ni-assisted laser oxidation process. In Figure 3a, the XPS spectrum of W at the surface of the WSe_2_ film after the Ni-assisted laser oxidation process shows W^6+^ valence states of W 4f spectra with binding energies located at 37.3 and 35.1 eV. Figure 3b shows XPS depth profiles, with which the atomic ratios of W, O, and Se were calculated from Figure 3c,d. The atomic ratios of W and O, being 30% and 70%, were confirmed, which are consistent with the results of TEM-EDS line scan profiles (Figure S3). The sputtering rate was 24 nm/min measured with a Si/SiO_2_ standard sample. The evolution of the W 4f spectra after the Ni-assisted laser oxidation process (Figure 3c) shows that the W 4f_5/2_ (37.3 eV) and W 4f_7/2_ (35.1 eV) peaks of WO_3_ appear from surface to depth of 60 nm calculated from the sputtering rate while the evolution of the corresponding Se 3d spectra (Figure 3d) shows the disappearance of Se in whole layer after the Ni-assisted laser oxidation process. Note that the peaks of the W^4+^ valence state represent the remaining bonds of W and Se, providing additional evidence of the complete oxidation of WSe_2_ into WO_3_.^17^ As shown in Figure S4a, the XPS O 1s spectra of the WSe_2_ film before and after the Ni-assisted laser oxidation process clearly reveals the emergence of oxygen-related signals. Before laser irradiation, no detectable oxygen peak is observed, indicating the absence of oxidation. After the laser oxidation process, a distinct O 1s peak appears at 529.4 eV, corresponding to the lattice oxygen of WO_3_. In contrast, Figure S4b presents the O 1s signal from the underlying Al_2_O_3_ layer, located at 530.8 eV. The clear distinction between these two peaks confirms the successful formation of WO_3_ from WSe_2_ after the Ni-assisted laser oxidation process. Furthermore, the binding energies of W 4f and Se 3d spectra for the WSe_2_ film before the Ni-assisted laser oxidation process were measured, as shown in Figure S5. The W 4f_5/2_ and W 4f_7/2_ peaks corresponding to the W^4+^ state are located at 33.4 and 31.2 eV, respectively. Meanwhile, the Se 3d_2/3_ and Se 3d_2/5_ peaks associated with Se^2–^ appear at binding energies of 54.1 and 53.3 eV. These signals remain consistent from the surface to a deeper depth of 60 nm, confirming the complete WSe_2_ film before the Ni-assisted oxidation process. The correlative results from TEM, TEM-EDS line scan, and XPS affirm that the Ni-assisted laser oxidation process enables spatially selective and chemically distinct oxidation of WSe_2_ into WO_3_.

**Figure 3:**
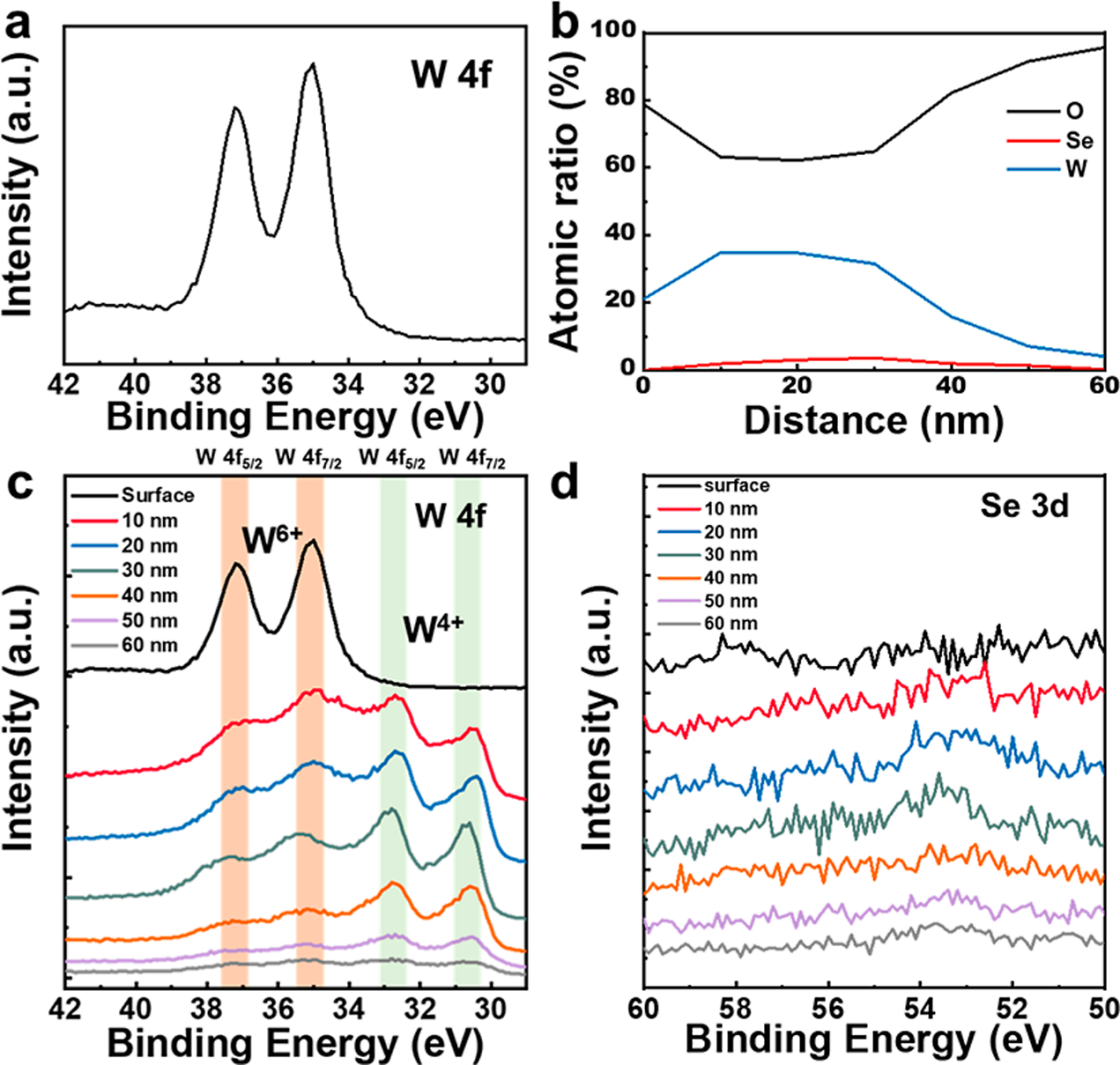
(a) Binding energy of W 4f on the surface of the laser spot. (b) XPS atomic ratio depth profile after laser oxidation and binding energy spectra of (c) W 4f. (d) Se 3d.

Furthermore, the impact of oxygen flux during the Ni-assisted oxidation process was investigated. The OM image shows a high contrast of a circular pattern formed after the Ni-assisted oxidation process, which is similar to the results, as shown in Figure 2a, and the corresponding Raman spectra show the peak of WO_3_ formed by the Ni-assisted oxidation process with the higher oxygen flux than the previous experiment (Figure 4a,b). The XPS and TEM analyses were utilized to investigate the structure of the WSe_2_ film after the Ni-assisted oxidation process under the higher oxygen flow rate (100 sccm). The binding energy spectra of W 4f verify the compositions of each W valence state, with the depth of the film increased by applying the Ar^+^ etching process. The Se 3d peak indicates the remaining amount of WSe_2_ in the whole structure (Figure 4c,d). The evolution of W 4f spectra shows higher W^4+^ peaks at 30.8 and 33 eV, and Se peaks at 53.1–54 eV, with a depth of 60 nm (Figure 4d). Interestingly, despite the increase in the supply of O_2_, the thickness of the WO_3_ oxide after the Ni-assisted oxidation process was observed to be thinner to that formed under the lower O_2_ flow rate with identical Ni-assisted oxidation process (60 s and 15 W). The rapid formation of the WO_3_ layer at the surface of the WSe_2_ film under a high oxygen flow rate likely suppresses further oxidation by blocking diffusion of oxygen into the underlying WSe_2_ film. Such self-limiting oxidation behavior results in a thinner WO_3_ film after the Ni-assisted oxidation process.^29^ The cross-sectional TEM image and the corresponding EDS line scanning results, as shown in Figure 4e,f, reveal that the resulting WO_3_ layer is thinner compared to that formed under the low O_2_ flow rate condition. This observation highlights a key kinetic difference between the oxidation process under low O_2_ flow rate and O_2_-rich environments. While low O_2_ flow rate allows more sustained oxygen diffusion into the film, high-flow conditions promote rapid surface passivation. These findings imply that the oxide layer thickness is not only governed by oxygen availability, but also by the interfacial kinetics and structural integrity of the initially formed WO_3_. Thus, tuning the oxygen flow rate offers a feasible pathway to systematically control the oxide film morphology and thickness.

**Figure 4:**
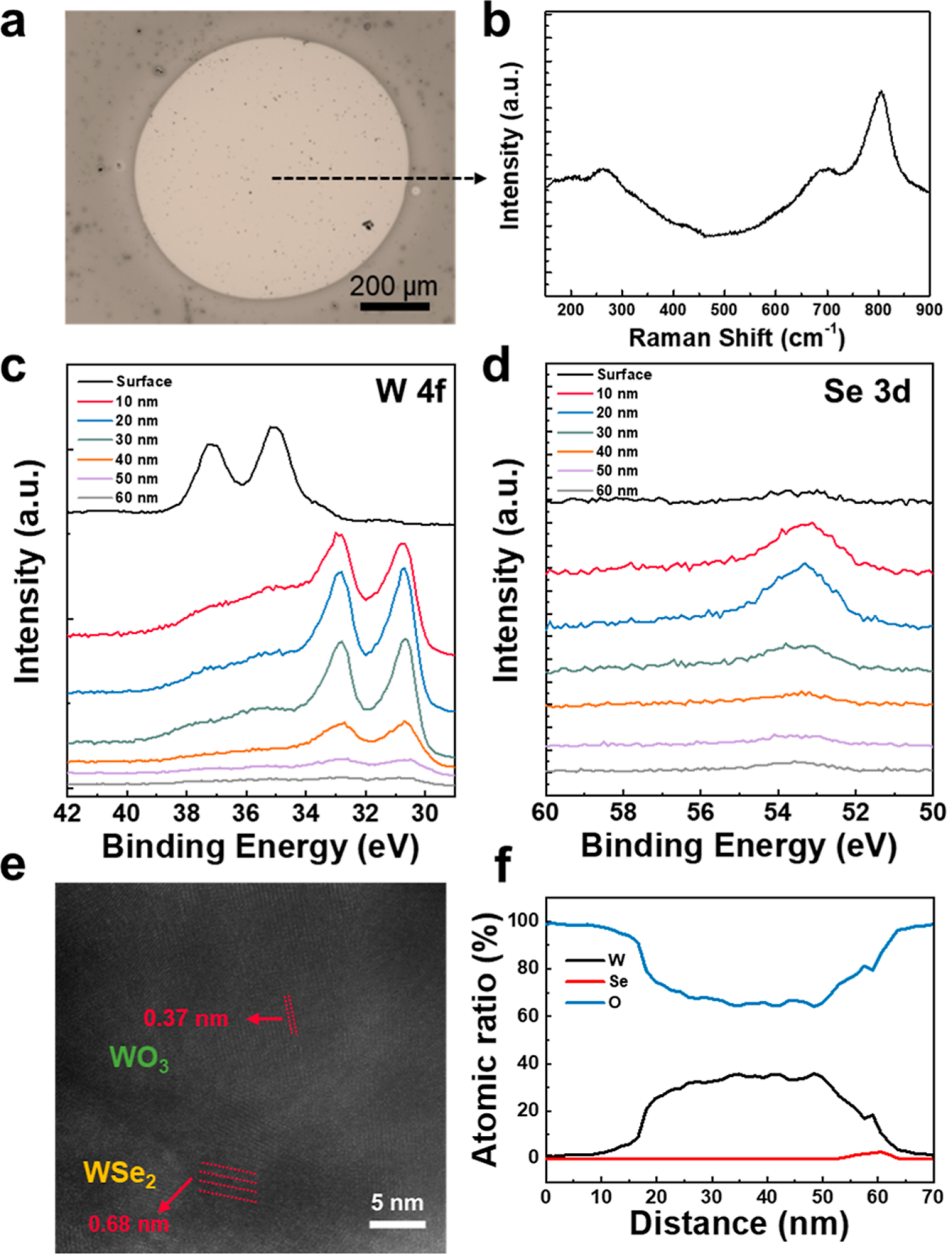
(a) Optical image of the Ni/Al_2_O_3_/WSe_2_ film applied with a CW laser under a high O_2_ flow rate. (b) A Raman spectrum of WO_3_ formed by the laser oxidation under a high O_2_ flow rate. XPS depth profile of WO_3_/WSe_2_ film formed by laser oxidation under high O_2_ flow rate. Binding energy spectra of (c) W 4f and (d) O 1s. (e) Cross-section TEM image of the film formed by laser oxidation under high O_2_ flow rate. (f) TEM-EDS line scan profiles on the laser oxidized WO_3_ structure.

Since the formation of the WO_3_ film can be precisely controlled by the underlying Ni layer after the Ni-assisted oxidation process, it allows us to precisely form different patterns of the WO_3_ layers, depending on how we create the patterns of the underlying Ni layer by lithography methods, including photolithography or e-beam lithography. It means that the dimension of the laser beam will not limit the spatial resolution of the WO_3_ layer, while it will be limited by the resolution of the underlying Ni layer. To demonstrate this concept, patterned Ni layers were prepared by photolithography to form an identical patterned WO_3_ layer from the WSe_2_ film after the Ni-assisted oxidation process. Here, Raman mapping images were conducted across the boundary between the underlying Ni-deposited and the non-Ni-deposited regions, followed by the Ni-assisted laser oxidation process. An OM image (Figure 5a) shows the color contrast, indicating the formation of WO_3_ oxidized from the WSe_2_ film on the Ni-deposited regions after the Ni-assisted laser oxidation process. At the same time, there is no color change on the WSe_2_ area without the underlying Ni-deposited regions after the Ni-assisted laser oxidation process. The corresponding Raman mapping image (Figure 5b) shows the mapping intensity of the WO_3_ characteristic peak (∼801 cm^–1^), which is significantly enhanced in the region with the underlying Ni layer deposited below Al_2_O_3_ and WSe_2_ films. In contrast, the areas without the Ni-deposited layer show no WO_3_ signals under the identical Ni-assisted laser oxidation process. The diameter of the laser spot is about 700 μm, which is larger than the size of the OM image in Figure 5a (20 × 20 μm^2^), indicating that the laser source should irradiate the whole area. This spatial contrast strongly supports that the assistance of Ni promotes localized oxidation of WSe_2_. To further confirm the effect of Ni heating on the laser oxidation process, we applied the highest power (27 W) of the CW laser to the samples without a Ni layer under the Al_2_O_3_/WSe_2_ film. The OM image shows no damage after the laser irradiation (Figure S6a). The corresponding Raman spectrum reveals that there is only a WSe_2_ signal at 250 cm^–1^, and does not show any WO_3_ peak in Figure S6b. The Ni layer likely acts as a laser-absorbing layer or facilitates thermal conduction during the laser irradiation, thereby enhancing oxidation kinetics in its vicinity. In addition, we prepared Ni patterns with “NTHU” by a photolithography method to prove that the Ni-patterned regions confined the formation of the WO_3_. The high contrast Raman mapping images of the NTHU pattern after the Ni-assisted laser oxidation process are shown in Figure 5c. The successful formation of a well-defined “NTHU” pattern through spatially controlled laser oxidation further highlights the high patterning resolution and selectivity of the proposed Ni-assisted method. Such capability demonstrates not only the scalability and compatibility of the process with device-relevant geometries but also its potential for localized functionality in future optoelectronic and sensing applications. The schematic diagram of the mechanism for the Ni-assisted laser oxidation process in this work is presented in Figure 5d1 to d3. Since the absorbance of Al_2_O_3_ and WSe_2_ at the laser wavelength of 808 nm is much lower than that of the Ni layer, the CW laser is mainly absorbed by the Ni layer (Figure 5d1). As the heat from Ni layer transfers upward to the WSe_2_ layer, the O_2_ gas in the chamber reacts with the WSe_2_ layer, forming WO_3_ (Figure 5d2). The transformation process can be understood in terms of a localized photothermal mechanism. Upon laser irradiation, the Ni layer efficiently converts optical energy into thermal energy due to its high absorption and moderate thermal conductivity. The generated heat is confined at the Ni layer, resulting in a steep temperature gradient across the vertical stack. This localized heating raises the interfacial temperature above the activation energy required for oxidation, while the surrounding areas remain below this threshold. Consequently, the oxidation of WSe_2_ to WO_3_ occurs only at the Ni-covered regions, providing high spatial selectivity. During the process, the Se atoms in WSe_2_ are replaced by oxygen supplied from the O_2_ gas. The reaction can be described as a thermally activated anion-exchange process, where the local temperature rise promotes the diffusion of oxygen species into the WSe_2_ lattice and the outward migration of selenium. This results in the formation of a WO_3_ layer with a thickness determined by the balance between thermal diffusion and oxidation kinetics. Moreover, the presence of the Ni layer not only defines the heat distribution but also stabilizes the interfacial temperature, minimizing lateral thermal diffusion and structural damage. As shown in Figure 5d3, the oxidation boundaries are confined to the Ni-patterned regions, confirming the spatially selective and energy-efficient nature of the Ni-assisted laser oxidation process. This mechanism underlies the controllability and reproducibility of the transformation, distinguishing it from conventional laser oxidation methods that often suffer from uncontrolled heat spreading. Besides the influence of the underlying Ni layer, the selection of the substrates is also another critical issue for this oxidation method. In whole experiments, quartz substrates were selected for the laser oxidation experiments due to their low thermal conductivity, optical transparency, and chemical inertness. Compared to other substrates, such as SiO_2_/Si substrate, quartz enables more efficient heat localization during the laser irradiation process. If the SiO_2_/Si substrate was used, the Si will absorb the CW laser, which is not suitable for the Ni-assisted laser oxidation process.^30,31^ The OM image shows that the film gets destroyed, and the corresponding Raman spectrum shows no WO_3_ formation after the laser oxidation process, as shown in Figure S6c,d. The high thermal conductivity of Si limits the efficiency of laser-induced thermal oxidation by rapidly dissipating heat away from the WSe_2_ surface. In addition, the nontransparent nature of silicon precludes the backside laser irradiation. These factors may impose constraints on oxidation uniformity and cause failures on patterning applications.

**Figure 5:**
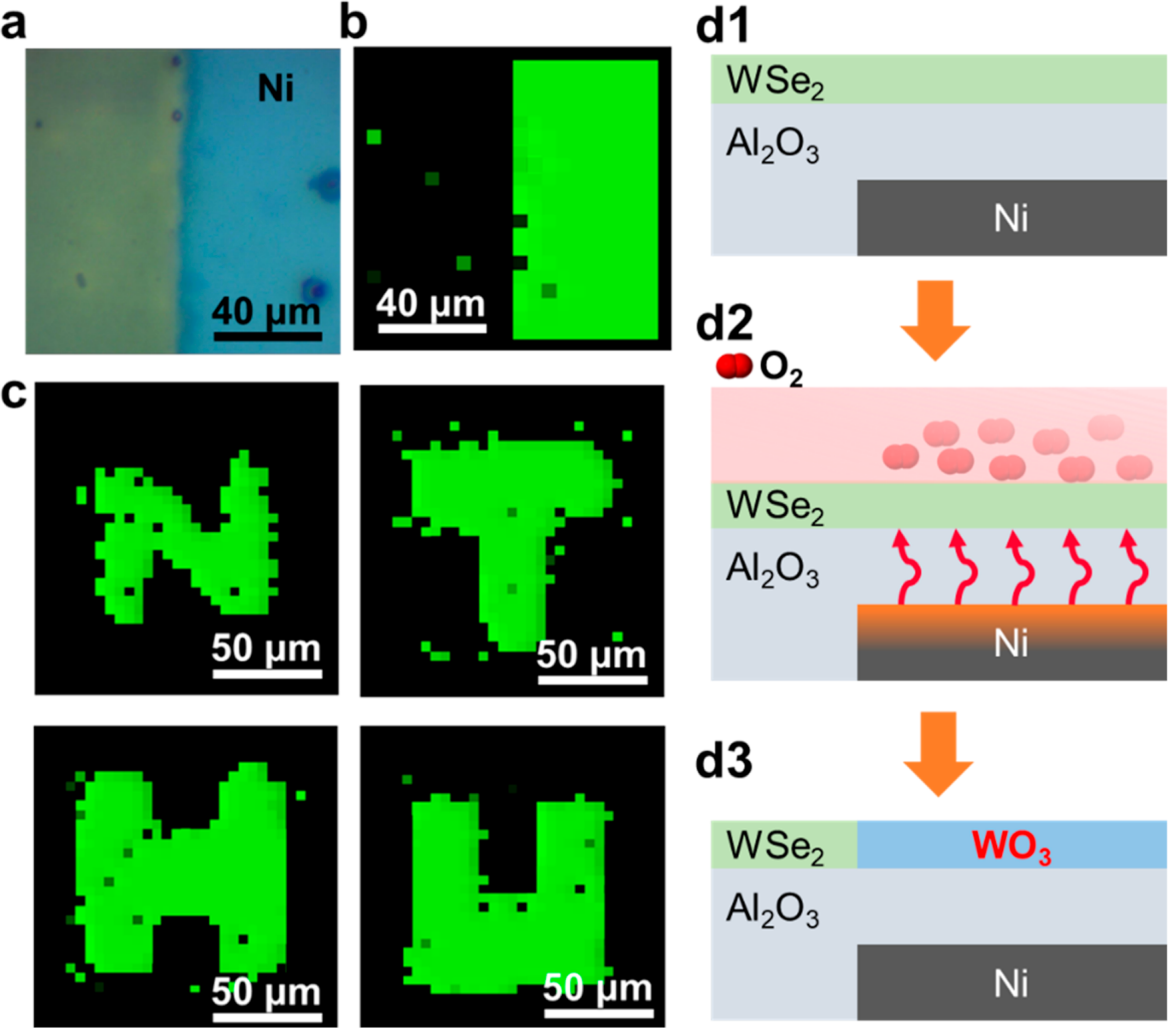
(a) OM image and (b) Raman mapping of WO_3_ peak position at the edge of Ni after laser oxidation. (c) Raman mapping images of the WO_3_ peak on the NTHU patterns. (d) Schematic diagram of the mechanism of the Ni-assisted laser oxidation process.

The ability to locally induce the formation of the WO_3_ film via the patterned-Ni layers enables the creation of functional domains with distinct electrical and optical properties.^23,32^ To evaluate the practical applications of such selectively oxidized regions, we fabricated the Ni/Al_2_O_3_/WO_3_, Ni/Al_2_O_3_/WSe_2_, and Ni/Al_2_O_3_/laser-oxidized-WO_3_ stackings into RRAM structures and compared their electronic transport characteristics. As shown in Figure 6a–c, the Ni layer was used as the bottom electrode, and the W electrode was deposited by e-beam deposition as the top electrode for the final step of device preparation. Figure 6a shows the current–voltage (I–V) switching characteristics of devices, which corresponds to the Ni/Al_2_O_3_/laser oxidized-WO_3_/W structure in the inset of Figure 6a. The high resistive state (HRS) and low resistive state (LRS) of the device were observed, with a set voltage of approximately −20 V, and a reset voltage close to 20 V under the current limit at 0.01 A. The on/off ratio can be observed to be 100 at the operating voltage of 1 V for the Ni/Al_2_O_3_/laser oxidized-WO_3_/W structure. The set and reset changes observed in the I–V curves indicate a filament-type switching mechanism. The I–V curve in Figure 6b was measured from the Ni/Al_2_O_3_/WSe_2_/W structure, where the laser oxidation process is not applied to the WSe_2_ layer. There is no switching behavior observed in the structure since the Al_2_O_3_ layer after the Ni-assisted laser oxidation process does not support the formation of the filamentary switching or the charge-trapping behaviors under the applied bias conditions. The possible reason is that the thermal treatment in oxygen-rich conditions may have passivated oxygen vacancy sites within the Al_2_O_3_ layer, suppressing the formation of conductive filaments typically required for resistive switching in RRAM devices.^33^ Additionally, the interface between Al_2_O_3_ and WSe_2_ may lack the defect states or electrochemical activity needed to sustain the bistable resistance state. To further confirm the benefits of the Ni-assisted laser oxidized WO_3_ for RRAM performance, we prepared the Ni/Al_2_O_3_/WO_3_/W sample by e-gun deposition without any annealing process. The I–V characteristic shows no obvious set or reset phenomenon that represents RRAM behavior in Figure 6c. These comparative results demonstrate that neither pristine WSe_2_ nor e-gun-deposited WO_3_ layers alone exhibit significant switching behavior. Note that only the laser-oxidized WO_3_ structures exhibit RRAM characteristics, confirming the importance of controlled oxidation pathways and heterointerface engineering in enabling device functionality.

**Figure 6:**
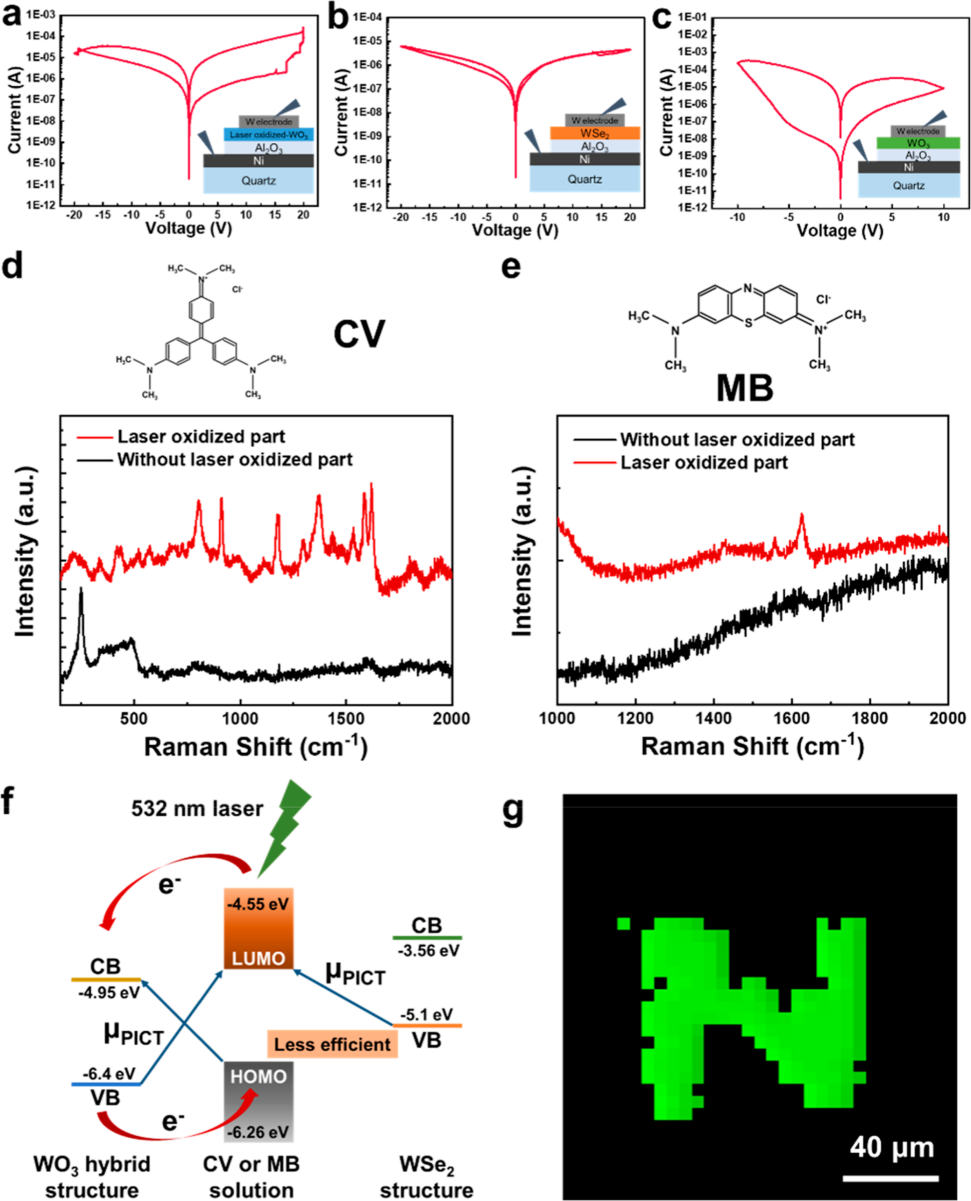
Current–voltage (I–V) characteristics of the forming process of the devices (a) with the laser irradiation process, (b) without the laser irradiation process, and (c) the WO_3_ film without selenization and laser irradiation process. (d) Raman spectra of CV on the laser-oxidized WO_3_ sample and (e) Raman spectra of MB on the laser-oxidized WO_3_ sample. (f) Schematic diagram of the SERS mechanism on WO_3_ and WSe_2_ structures.[32] Raman mapping of the WO_3_ pattern at the peak of 808 cm^–1^ after SERS measurements.

Beyond its advantages on RRAM applications, the laser-oxidized WO_3_ structure also exhibits promising optical properties that enable its use in sensing applications. To assess the potential of the laser-oxidized WO_3_ structures for sensing applications, surface-enhanced Raman scattering (SERS) measurements were conducted using crystal violet (CV) and methylene blue (MB) as sensing molecules. As shown in Figure 6d, the Ni-assisted laser-oxidized WO_3_ exhibits strong and well-defined Raman peaks corresponding to CV molecules with a concentration of 10^–6^ M, in contrast to the WSe_2_ without applying the Ni-assisted laser oxidation process, which does not show Raman signals of CV molecules. Moreover, Figure 6e presents a similar result using MB molecules (10^–6^ M) as the Raman sensing target, revealing that the Ni-assisted laser-oxidized WO_3_ structure exhibits strong and well-defined Raman peaks of MB molecules, further validating the generality of the enhancement effect. It should be noted that the excitation laser power is a critical parameter in determining the SERS enhancement performance. At low excitation powers, the local electromagnetic field is insufficient to produce a strong Raman signal, resulting in poor signal-to-noise ratios and higher detection limits. Increasing the excitation power leads to a proportional increase in Raman intensity due to stronger plasmon-molecule coupling and enhanced photoinduced charge transfer between the analyte and the WO_3_ domains. The consistent signal amplification observed across different molecules suggests that the SERS enhancement is not molecule-specific but rather originates from the intrinsic electronic and surface properties of the Ni-assisted laser-oxidized WO_3_. Figure 6f presents a schematic illustration of the proposed SERS enhancement mechanism between the Ni-assisted laser-oxidized WO_3_ and WSe_2_ without the Ni-assisted laser oxidation structures. The observed SERS enhancement can be attributed primarily to a charge transfer mechanism, given the dielectric nature of WO_3_.^32^ Specifically, the energy levels of WO_3_ align favorably with the highest occupied molecular orbital (HOMO) and the lowest unoccupied molecular orbital (LUMO) of the probe molecules such as crystal violet (CV) and methylene blue (MB). This band alignment facilitates photoinduced electron transfer between the WO_3_ substrate and the adsorbed molecules under laser excitation, thereby increasing the polarizability of the molecules and enhancing their Raman scattering cross-section. Notably, the Raman mapping image of the WO_3_ peak at 801 cm^–1^ in Figure 6g was obtained after CV exposure and SERS measurements, yet the characteristic oxide peaks remain visible and spatially confined. This result indicates that the laser-induced WO_3_ regions are chemically stable and structurally robust, withstanding molecular adsorption. The ability to spatially pattern SERS-active domains via the Ni-assisted laser oxidation process not only demonstrates structural control but also aligns with current efforts in developing multiplexed and addressable sensing platforms. Patterned SERS substrates have emerged as a powerful tool for location-specific molecular detection, enabling on-chip and CMOS-compatible applications in biosensing and environmental monitoring.^34^ These results show that Ni-assisted laser oxidation not only enables spatially defined patterning of oxide domains but also imparts them with functional electrical and optical properties.

## Conclusion

In summary, we have demonstrated a Ni-assisted laser oxidation approach for spatially controlled transformation of WSe_2_ film into WO_3_ film by the high optical absorbance of Ni at 808 nm, the localized heating during the laser irradiation facilitates efficient oxidation of WSe_2_ under controlled oxygen atmospheres. Structural characterizations such as OM, Raman, AFM, TEM, and XPS confirm the formation of crystalline WO_3_ within the irradiated regions. Besides, the Ni-assisted laser oxidation process exhibits spatial selectivity, as evidenced by patterned oxidation confined to Ni-deposited areas. Furthermore, the oxidation behavior is strongly influenced by oxygen flow rate. Under high oxygen conditions, a self-limiting oxidation phenomenon occurs, where a rapidly formed WO_3_ surface passivates further reaction, leading to thinner oxide layers. This observation shows the critical role of interfacial kinetics and initial oxide structure in determining final film morphology. Functionally, the Ni-assisted laser-oxidized WO_3_ structures exhibit enhanced electrical performance with resistive switching characteristics, demonstrating their viability for RRAM applications. In addition, the laser oxidized regions show significant SERS activity toward molecules such as CV and MB, enabling ultrasensitive molecular detection. The SERS enhancement can be attributed to charge-transfer mechanisms facilitated by oxygen vacancies and the hybrid interface between WO_3_ and WSe_2_. Overall, this work provides a scalable and versatile platform for engineering oxide structures through metal-assisted laser oxidation, offering new possibilities in device fabrication, photonic sensing, and multifunctional material integration.

## Experimental Section

### Synthesis of Ni/Al_2_O_3_/WSe_2_ Stacking Films

Ni (50 nm), Al_2_O_3_ (50 nm), and WO_3_ (40 nm) layers were deposited on a quartz substrate by e-beam evaporation in order. Before e-beam evaporation, the quartz substrate was cleaned in a sequence of acetone, isopropyl alcohol, and deionized water. After the deposition of a Ni layer, the sample was placed at the middle stage in the chamber of the vertical selenization furnace, and selenium (Se) granules were placed in the top container of the selenization furnace. After introducing the sample and Se granules, the vertical tube was first pumped under a pressure of 9 × 10^–3^ Torr. Then, a mixed carrier gas of N_2_ (50 sccm) and H_2_ (100 sccm) was introduced from the top side of the furnace to carry the Se gas downward. The furnace was heated to 450 °C and maintained at the same temperature during the synthesis process with a fixed gas flow. When the temperature reached 450 °C, the plasma was opened at the same time and kept at a power of 150 W during the synthesis process. After the process, the H_2_ gas and plasma were turned off, and the chamber was naturally cooled down to 80 °C. Then, the chamber was vented to cool down to room temperature, allowing the sample to be removed from the stage.

### Laser Oxidation Process

The WO_3_ structure was synthesized by a laser system with a continuous wavelength of 808 nm. The sample was transferred to a chamber, and the pressure of the chamber was pumped to below 1 × 10^–2^ Torr. O_2_ gas was introduced to the chamber when it reached the set pressure. After the pressure stabilized, the laser was applied to the sample, reacting with the O_2_ gas to convert WSe_2_ into WO_3_.

### Material Characterization

Raman spectra and mapping images of the WSe_2_, WO_3_ film, and CV, MB molecules for SERS were acquired on the Andor Kymera 328i spectrograph with a 532 nm wavelength excitation laser, and the beam size of the laser is 2 μm with a 100× objective lens. The surface morphology of samples before and after laser irradiation was analyzed by AFM (Bruker, Dimension Icon). The chemical compositions, chemical bonding, and electronic structures were determined using XPS (PHI 5000 Versaprobe II), and the XPS spectra were calibrated by the binding energy of the C 1s peak at 284.5 eV. HRTEM analyses were carried out to examine the atomic structure of WSe_2_, WO_3_, WSe_2_, and WO_3_ hybrid layers by TEM with the Cs-corrector (JEOL JEM-ARM200F).

### Device Fabrication and Electrical Measurements

A thickness of 80 nm tungsten (W) was deposited by e-beam evaporator on the WSe_2_, e-gun deposited WO_3_, and laser-oxidized WO_3_ film as the top electrode. The bottom Ni layer served not only as the heating layer but also as the bottom electrode for the RRAM device. Moreover, the Al_2_O_3_ layer was served as the switching layer. The switching characteristics were measured by an Agilent B1500A Semiconductor Device Parameter Analyzer in a probe station.

## Supplementary Material



## Notes

Process flow of WSe_2_ fabrication and formation of WO_3_ film by the Ni-assisted laser oxidation process; optical image and corresponding Raman spectra of the Ni/Al_2_O_3_/WSe_2_ film applied with the highest laser power; the laser oxidation results obtained under different laser power and laser irradiation times with laser line-scan; the laser oxidation results acquired under different laser power and laser irradiation times with 100 nm Al_2_O_3_; TEM-EDS line scan profile on laser oxidized WO_3_ structure; binding energy of W 4f and Se 3d before laser illumination; optical image and Raman spectra of the WSe_2_ film without Ni absorption layer after laser illumination; optical image and Raman spectra of the WSe_2_ film with laser irradiation applied on Si/SiO_2_ substrate (PDF)
