# Supplementary material for: Nickel-Assisted Laser Oxidation of WSe2 Layered Films for Spatially Controlled WO3 Patterning Process toward Resistive Memory and Molecular Sensing
Source: ACS Appl Mater Interfaces. 2025 Dec 1;17(49):67272–83. doi: 10.1021/acsami.5c17500 (PMC12874381; doi:10.1021/acsami.5c17500)
Supplement: Supplementary file 1 [file am5c17500_si_001.pdf]

Nickel-Assisted Laser Oxidation of WSe_2 Layered Films for Spatially Controlled WO_3 Patterning Process toward Resistive Memory and Molecular Sensing

Yu-Chieh Hsu[1,2], Ruei-Hong Cyu[1,2], Yu-Qi Huang[1,2], Chieh-Ting Chen[1,2], Po-Chien Lai[1,2], Yi-Jen Yu[5], Chang-Hong Shen[2], and Yu-Lun Chueh [1,2,3,4*]

[1]Department of Materials Science and Engineering, National Tsing-Hua University, Hsinchu 30013, Taiwan

[2]College of Semiconductor Research, National Tsing-Hua University, Hsinchu, 30013, Taiwan

[3]Department of Physics, National Sun Yat-Sen University, Kaohsiung, 80424, Taiwan

[4]Department of Materials Science and Engineering, Korea University, Seoul 02841, Republic of Korea.

[5]Instrument Center, National Tsing Hua University, Hsinchu, 30013, Taiwan

[*]E-mail: ylchueh@mx.nthu.edu.tw

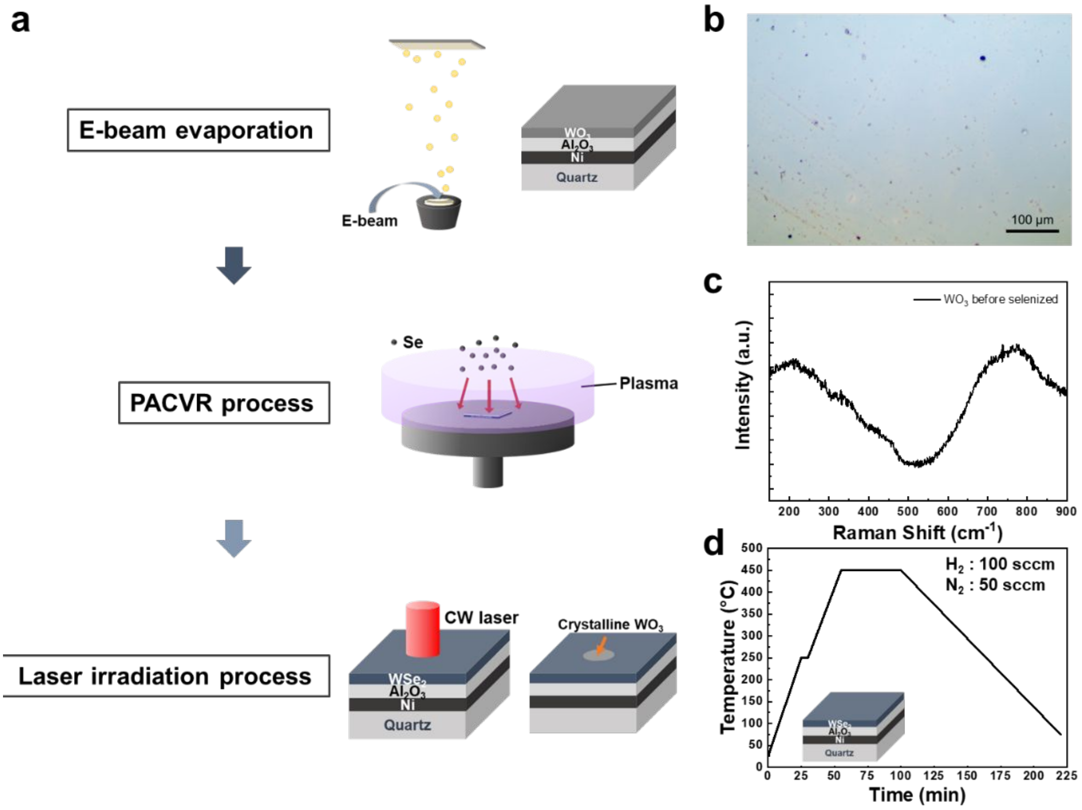

Figure S1 (a) Process flows of WSe_2 fabrication and synthesis of WO_3 film by a Ni-assisted laser oxidation process. (b) An optical image of the WO_3 film after E-beam evaporation. (C) A Raman spectrum of WO_3 film after E-beam evaporation. (d) The heating and cooling curve of WSe_2 film synthesized by the PACVR process.

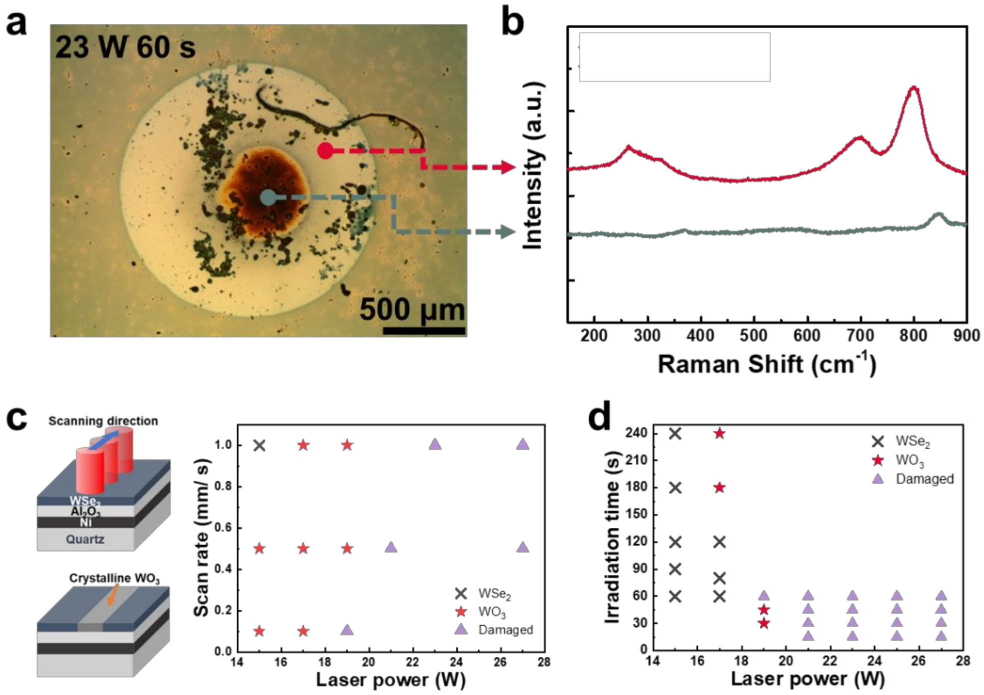

Figure S2 (a) An optical image of the Ni/Al_2O_3/WSe_2 film applied with the highest laser power. (b) Raman spectra corresponding to the place marked in (a). (c) The laser oxidation results obtained under different laser power and laser irradiation times with the laser line-scan. (d) The laser oxidation results acquired under different laser power and laser irradiation times with 100 nm Al_2O_3.

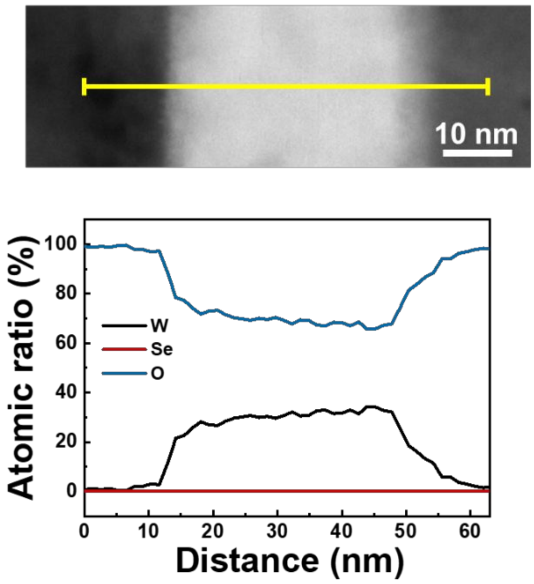

Figure S3 TEM-EDS line scan profiles on the laser-oxidized WO_3 structure.

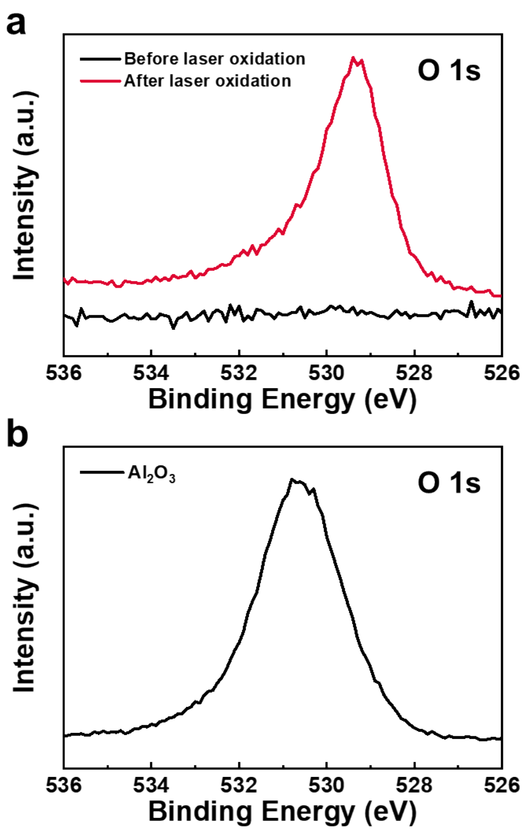

Figure S4 Binding energy of O 1s (a) before and after laser illumination on WSe_2 film. (b) Binding energy of O 1s of Al_2O_3 layer.

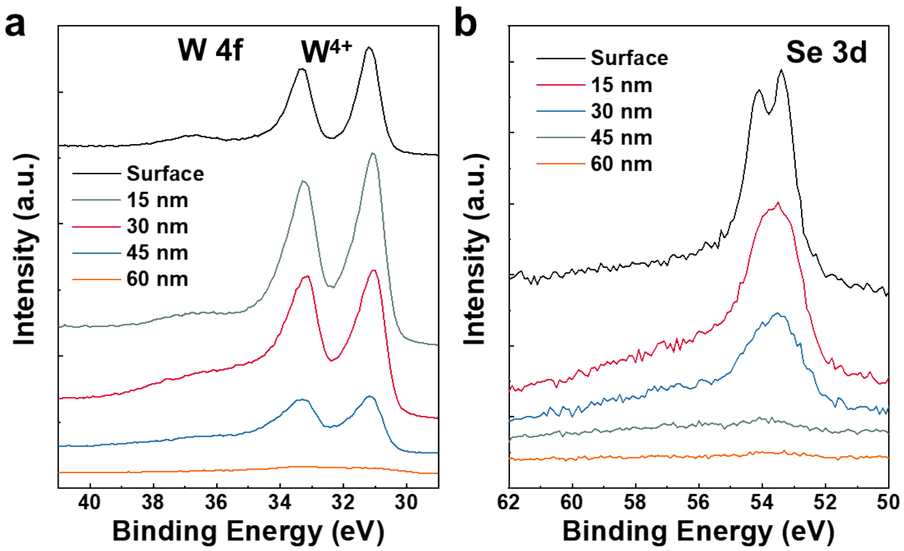

Figure S5 Binding energy of (a) W 4f and (b) Se 3d before laser illumination.

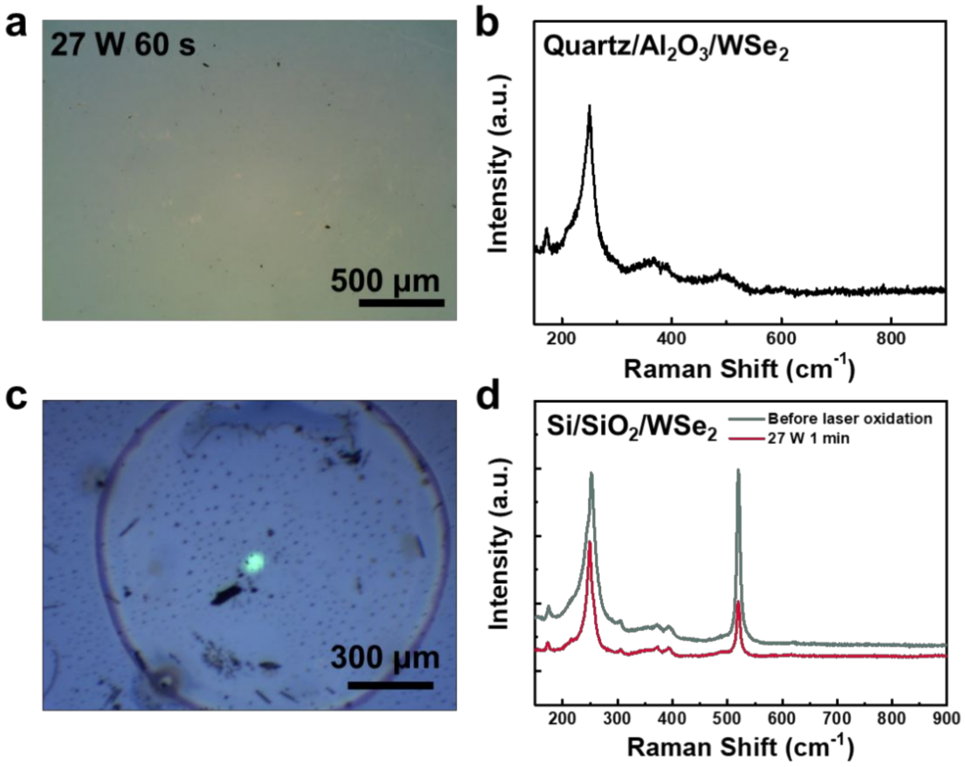

Figure S6 (a) Optical image and (b) Raman spectra of the WSe_2 film without a Ni absorption layer after laser illumination. (c) Optical image and (d) Raman spectra of the WSe_2 film with laser irradiation applied on a Si/SiO_2 substrate.
